# Scratch Card Near-Miss Outcomes Increase the Urge to Gamble, but Do Not Impact Further Gambling Behaviour: A Pre-registered Replication and Extension

**DOI:** 10.1007/s10899-020-09932-7

**Published:** 2020-02-25

**Authors:** Madison Stange, Mike J. Dixon

**Affiliations:** Department of Psychology, University of Waterloo, Waterloo, ON N2L 3G1 Canada

**Keywords:** Scratch cards, Near-misses, Purchasing behaviour, Erroneous cognitions, Urge to gamble

## Abstract

Scratch card near-misses, outcomes in which two out of three required jackpot symbols are uncovered, have been shown to erroneously increase the urge to continue gambling. It remains unknown if and how these outcomes influence further gambling behaviour. Previous studies examining the influence of near-misses on purchasing behaviour offered a low-stakes gamble to participants after experiencing a near-miss or a regular loss. We sought to investigate the influence of these outcomes on scratch card purchasing behaviour with a stronger test of participants’ gambling behavior by having them either “cash out” or risk all of their winnings to purchase another card. Additionally, we sought to test an original hypothesis that endorsement of the illusion of control might influence the decision to purchase additional scratch cards. We pre-registered our hypotheses, sample size, and data analysis plan. 138 subjects experienced two custom-made scratch card games that included a win on the first card (for all participants) and either a regular loss or a near-miss in the final outcome position on the second card (between-subjects manipulation). Although near-miss outcomes increased the urge to continue gambling relative to regular losses, no differences in the rates of purchasing were found between the conditions. Additionally, no support for our hypotheses concerning the influence of the illusion of control in near-miss outcomes was found. These results are discussed in terms of previous studies on scratch card gambling behaviour and subjective reactivity.

## Introduction

Despite their wide appeal and ubiquitous availability, how gamblers experience scratch card lottery games remains an understudied area of gambling research. Existing research has focused on specific populations in which scratch card gambling may be of concern, specifically youth and minors who access scratch cards despite legal age restrictions and for whom scratch cards are a popular gambling activity (Felsher et al. 2004a, b; Wood and Griffiths 1998). While harm derived uniquely from scratch cards is estimated to be low (Hendriks et al. 1997), evidence suggests that frequency of scratch card gambling positively predicts problem gambling severity (Short et al. 2015; Stange et al. 2018). Additionally, case studies of pathological scratch card gambling have been reported in the literature (Raposo-Lima et al. 2015). These findings suggest that scratch card games may not be as innocuous as most people believe them to be. Indeed, specific scratch card features have garnered concern from gambling researchers, including their continuous nature, rapid event frequency, and low price point (Griffiths 2002)—features which may lead some gamblers to purchase additional scratch cards with their winnings, or conversely, to chase their losses. In line with these concerns, an observational study of naturalistic scratch card gambling found evidence of episodic loss chasing and that scratch card gamblers were more likely to chase their losses on lower-denomination scratch cards (Whiting et al. 2016).

Another concerning feature of scratch cards is that of the near-miss—a special type of losing outcome that falls *just* short of a jackpot prize (Reid 1986). Such outcomes have been most extensively studied in slot machine games, where they have been shown to increase physiological arousal and frustration (Dixon et al. 2011, 2013), recruit reward related brain areas (Clark et al. 2009), and lead to persistence in slot machine gambling behaviour (Kassinove and Schare 2001; Côté et al. 2003), despite being a monetary loss. Although scratch card near-misses had been described in the literature (Moran 1979; Reid 1986; Wood and Griffiths 1998), only recently has experimental research been undertaken to understand the impact that these types of outcomes have on the experience of scratch card gambling and subsequent gambling behaviour.

Existing experimental research on scratch card games has focused on the psychophysiological and cognitive experience of various scratch card outcomes (Stange et al. 2016, 2017a). In these experiments, which utilize custom-made scratch card games, participants are hoping to uncover three matching symbols within a game matrix of six symbols (see Stange et al. 2016). In this type of game, a near-miss consists of uncovering two of the three required symbols to win a top prize, a win consists of uncovering three matching prize symbols, and a regular loss consists of six unique, non-matching symbols. Gamblers tend to experience greater physiological arousal (as measured by skin conductance and heart rate) leading up to the reveal of winning and near-miss scratch card outcomes, relative to regular losses (Stange et al. 2016, 2017a). Additionally, scratch card near-miss outcomes are consistently rated as significantly more frustrating, subjectively arousing, disappointing, and urge inducing than regular losses (Stange et al. 2017a). It is important to remember that although near-miss outcomes are capable of evoking such reactions in gamblers, they are *objectively* equivalent to regular losses. Therefore, despite their objective value, gamblers react differently to these near-misses than other non-winning outcomes.

Scratch card games have other unique structural features, such as their relatively low price point. The financial accessibility of these games, combined with the arousal and urge-inducing effects previously discussed could allow near-miss outcomes to influence decision making about whether to stop or continue gambling. Recently, Stange et al. (2017b) examined purchasing behaviour following both near-miss (two out of three matching top prize symbols) and regular losing outcomes (six non-matching symbols) in scratch card games. In this paradigm, all participants initially received two scratch cards, and won $5.00 on their first card. On their second card, half of the participants experienced a near-miss on their final outcome, while the other half experienced a regular loss in the same position. Following these two cards, participants were asked if they would like to purchase an additional card with their winnings (at a cost of $2.00). Although participants rated the near-miss outcome as more urge inducing than the regular loss, there was no significant difference between the conditions in terms of rates of purchasing an additional card. However, a significant correlation between urge to continue gambling at the final outcome and the decision to purchase an additional card was found, but only for participants in the near-miss condition; no such relation was present in the loss condition. These results suggest that the urge to continue gambling induced by near-miss outcomes is associated with an individual’s decision to purchase additional scratch cards (Stange et al. 2017b).

In that study, some participants elected to purchase another card in the absence of an increase in the urge to gamble (i.e., in the loss condition; Stange et al. 2017b). This could have been due to the relatively low cost of purchasing the additional card (only $2.00) after having won $5.00 overall in the experiment. That is, if a participant decided to purchase an additional card they were only risking 40% of their total winnings for another chance at the top prize. Due to the low-stakes nature of this gamble, it is possible that a number of participants who purchased an additional card were doing so not because they felt particularly motivated per se (i.e., due to an increase in urge), but rather they purchased on a whim due to the low cost involved in this decision. In this study, urge ratings for purchasers in the loss condition were lower than urge ratings for purchasers in the near-miss condition, but there were only nominally more purchasers in the near-miss condition than in the loss condition (Stange et al. 2017b). Further, as previously discussed, a significant positive correlation between urge at the final outcome and purchase status was found *only* in the near-miss condition, suggesting that the decision to purchase an additional card is related to experienced urge following near-miss outcomes, but not losses. It is possible that making the decision to purchase an additional card riskier may reduce the number of participants who purchase in the absence of any increase in the urge to gamble. Additionally, having to risk all of one’s winnings in order to purchase another card is a common scenario in scratch card gambling. In most games the most likely “winning” prize is not in fact a true win, but what gamblers refer to as a *push*—a gain equivalent to a gamblers’ original wager, and equivalent to the price of another card.^1^ Here we sought to create a more ecologically valid test of participants’ gambling behavior following pushes, and subsequent regular losses and near-misses.

Although near-miss outcomes do reliably lead to increases in the urge to continue gambling (Stange et al. 2017a, b), the previously reviewed evidence suggests that the decision to purchase additional scratch cards is not entirely based on this subjective motivational state. We propose that this decision may reflect an interaction between momentary, state-level motivational processes and more stable, individual trait-level factors. A potential candidate for the latter is the illusion of control, a common gambling-related cognition (Langer 1975). As a construct, the illusion of control suggests a sense of agency over events that in actuality cannot be controlled, in the form of an inflated sense of personal skill (Langer 1975; Raylu and Oei 2004). In games of pure chance (e.g., scratch cards), each outcome is independent from the next. While near-miss outcomes are objectively losing outcomes, gamblers who endorse the illusion of control may see a near-miss as a signal of increasing skill (Sescousse et al. 2016) and believe that the *true* win may soon follow, should they continue to gamble (Reid 1986). Studies examining slot machine gambling have found that the desire to continue gambling following a near-miss is correlated with illusion of control scores (Billieux et al. 2012), although the illusion of control was not found to be a significant predictor of the desire to gamble after near-miss outcomes when included in a model with pleasure experienced after wins, social desirability bias, and endorsement of predictive and interpretive control over outcomes (all found to be significant predictors; Billieux et al. 2012). In another study examining erroneous cognitions and stop-button use in slot machine gambling, endorsement of skill-related cognitions regarding near-miss outcomes (e.g., “near-misses reflect my skill at this slots game and indicate that I was close to winning” and “near-misses indicate that a win is imminent”) were related to illusion of control scores (Dixon et al. 2018). These results suggest that endorsement of the illusion of control may play a role in the motivational impact of near-miss outcomes and, by extension, the subsequent decision to purchase additional scratch cards following them.

The current study had two central aims. The first was to investigate the influence of bet size and the endorsement of the illusion of control on the decision to purchase additional scratch cards, following both losing and near-miss outcomes. We predicted that a riskier gamble (a larger bet size involving 100% of the participants’ prior winnings) would lead to fewer participants purchasing in the absence of any sizable increase in urge (e.g., in the loss condition). Additionally, by potentially eliminating participants who make low-cost purchases on a whim (as in the former study), we sought to show that more participants in the urge inducing near-miss condition would purchase than participants in the loss condition. In terms of the illusion of control, we predicted that participants who purchase an additional scratch card following a near-miss outcome would score higher in endorsement of this erroneous cognition than participants who do not purchase an additional card following the near-miss. Finally, we predicted that the pattern of urge responses across the scratch card outcomes would replicate past findings, such that wins will be rated as significantly more urge inducing than regular losses, and that participants would report significantly more urge following the near-miss outcome than a regular losing outcome in the equivalent position (Stange et al. 2017b). We also predicted that previously observed associations between urge following the near-miss outcome and participants’ decision to purchase would be replicated.

The second aim of the current study was a more pragmatic one. As this study incorporates a replication attempt of results obtained with a relatively small sample size, we thought it imperative to replicate these findings with a larger sample. This, coupled with the inclusion of an original hypothesis to extend these findings, prompted us to pre-register our sample size, hypotheses, and data analysis plan in advance of data collection (registered on the Open Science Framework: https://osf.io/cbxrm). Although such practices are not currently universal in peer-reviewed addiction journals (Gorman 2019), pre-registered replications are extremely important given ongoing issues of reproducibility within psychological research (Open Science Collaboration 2015), and we believe are of utmost significance for investigations of addictive behaviours that have potential ramifications for clinical practice and potential policy changes.

## Method

### Participants

A sample of 138 undergraduate participants was recruited from the University of Waterloo Research Experience Group. All participants were prescreened to ensure that they were 18 years of age or older (the legal age to purchase scratch cards in Ontario), had experience with scratch card games, and were not in or had previously received treatment for problem gambling. One participant was excluded from all analyses due to a procedural error.

### Materials

#### Canadian Problem Gambling Index and Problem Gambling Severity Index

The Canadian Problem Gambling Index (CPGI) is a well-validated measure for assessing the frequency of specific gambling behaviors and gambling-related harm in the general population (Ferris and Wynne 2001). The CPGI contains the 9-item Problem Gambling Severity Index (PGSI), which screens for gambling harm and results in a numerical score ranging from 0 to 27 (the sum of all items). Participants respond to each item by stating how frequently the behaviour in question had applied to them over the last 12 months, with response options ranging from never (scored as 0), sometimes (1), most of the time (2), or almost always (3). Based on established criteria (Currie et al. 2013), PGSI scores can be used to categorize participants as non-problem (scores of 0), low-risk (scores of 1–4), moderate risk (scores of 5–7), or problem gamblers (scores of 8 or above). The CPGI and PGSI were administered to characterize our sample and were not analyzed further (the distribution of PGSI scores can be found in Table 1).

**Table 1:** Descriptive and demographic characteristics

Measure	Value
Age, mean (SD)	20.48 (1.95)
Gender, *n* (%)	
Female	108 (78.8)
Male	28 (20.4)
Gender queer/gender non-conforming	1 (0.7)
Frequency of scratch card gambling, *n* (%)	
1–5 times	114 (83.2)
6–10 times	18 (13.1)
11–15 times	3 (2.2)
16–23 times	1 (0.7)
24 or more times	1 (0.7)
Problem Gambling Severity Index, *n* (%)	
Non-problem gambling	86 (63.2)
Low-risk gambling	47 (34.6)
Moderate-risk gambling	2 (1.5)
Problem gambling	1 (0.7)

One participant did not submit data for the Problem Gambling Severity Index due to a technical error (and therefore counts for this measure will add to *N *= 136)

#### Gambling Related Cognitions Scale

The Illusion of Control subscale of the Gambling Related Cognitions Scale (GRCS; Raylu and Oei 2004) was administered to participants before the testing session in a survey of measures administered to the entire participant pool. This subscale consists of four items, each scored on a 7-point Likert scale, ranging from Strongly Disagree (1) to Strongly Agree (7).

#### Scratch Cards

The scratch card games that participants experienced during the experiment were similar to those used in previous investigations (Stange et al. 2016, 2017a). The custom-made scratch cards were called “Cash for a Month”, designed to emulate a popular card available for purchase in our home jurisdiction of Ontario, Canada. Each scratch card contained three scratch-off game squares, each containing six symbols. The goal of the game is to find three matching symbols within one game square. If three matching symbols are found, the participant wins the corresponding prize (all game symbols are prize amounts). The top prize of the game that was available to be won by participants was Cash for a Month, or $25 a week for 4 weeks (or a one-time payout of $100).

#### Measure of Gambling Urge

Participants rated their urge to continue gambling by responding to the following item: “How would you rate your desire to gamble on a scale from 0 (no desire to gamble) to 100 (overwhelming desire to gamble)?” (Young et al. 2008). The item was presented on a tablet computer (Lenovo TB-X103F) using a sliding scale with numerical anchors at 0 and 100.

### Design

The present study utilized a between-subjects design, such that participants were randomly assigned to experience either a regular loss (made up of no matching symbols within the game square) or a near-miss (consisting of 4 non-matching symbols and two top prize symbols within the game square) for their final outcome. All participants experienced a regular loss, a small win of $5.00, and another regular loss on their first card. On the second card, all participants experienced two regular losses before the between-subjects manipulated outcome in the final position.

Participants were randomly assigned to a condition based on the scratch cards that they chose. Scratch cards were housed in a display case that contained two removable trays which each held 48 scratch cards (a total of 96 scratch cards per display case). The first tray contained scratch cards with the following outcomes: a regular loss, a small win of $5.00, and another loss. The second tray contained a mixture of cards that contained either three regular losses or two regular losses and a near-miss. All participants chose a scratch card from both trays in the display case, ensuring both equal remuneration across participants and random assignment to condition.

After completing the first two scratch cards, participants were given the choice to purchase another card for $5.00. If participants decided to purchase, they chose an additional card from a second display case that contained two losses and a win of $5.00 (equating remuneration among participants). In both display cases, one top prize card was included within the array of cards.

### Procedure

Participants entered the lab room and were provided with an information letter outlining the details of the study. If participants chose to participate they provided written consent. Upon informed consent, participants completed the CPGI using a laptop computer (Lenovo ThinkPad model 4446-25U). Participants were then given instructions for the scratch card games. Participants were told that they would be starting with two scratch cards, that these first two cards would be free, and that they could potentially purchase another card at a later point in time, but that this would be explained later on in the study. The researcher introduced the game of Cash for a Month and showed the participant an example scratch card. Participants were told that to win on the scratch card game, they had to match three symbols within a given game square. The researcher explained that in order to win the top prize of Cash for a Month, corresponding to $25 a week for 4 weeks or $100 cash, participants needed to uncover three matching “MONTH” symbols within one game matrix, which would denote a top prize win. Participants were told that their odds of winning the top prize of the game were approximately 1 in 100. The researcher then informed participants that after each scratch card game, they would give a rating of their current desire to gamble on a scale from 0 to 100. The participant was shown an example of the desire to gamble item and the sliding scale used to indicate a response. The researcher instructed the participant to slide the indicator to the position on the scale that accurately reflected their current desire to gamble.

Participants selected their cards from the display case of scratch cards. The researcher removed the two trays of scratch cards from the display case and instructed the participant to choose one card from each tray. Once participants had chosen their scratch cards, the researcher directed them to a desk where they would scratch the cards.

The researcher inserted the first scratch card into a platform in front of the participant to ensure a consistent scratching experience between participants (see Stange et al. 2016). Participants were given a plastic scratching device to uncover the symbols. When participants had completed their first two scratch cards, the experimenter gathered the participant’s winnings ($5.00), placing a $5.00 bill on the display case. The researcher explained to the participant that since they had won $5.00 on their first card, that they could now purchase another card if they wanted. The researcher explained that they would be choosing a scratch card from a second display case of cards, but that their odds of winning the top prize of the game were the same, approximately 1 in 100. The researcher then asked the participant if they wanted to purchase another card.

If participants chose to purchase another card, the researcher took back the participants’ winnings ($5.00), and instructed the participant to choose one scratch card from any location in the second display case. When the participant selected a card, the experimenter again placed the card in the secure scratching platform and reminded the participant to fill out the desire to gamble items for each outcome on the tablet computer as they had for the first two scratch cards. After completing the third scratch card (if they elected to purchase) or after declining to purchase another card, participants were remunerated with the winnings from their scratch cards ($5.00 for purchasers and non-purchasers) and given a feedback letter outlining the details of the experiment as well as lottery-specific responsible gambling resources.

## Results

### Sample Characteristics

Age, gender, self-reported frequency of scratch card gambling, and PGSI scores are listed in Table 1. Participant’s scores on the PGSI were calculated according to established cut-off criteria (Currie et al. 2013).

### Purchasing Behaviour

Of the entire sample (*N* = 137), only 18% of participants chose to purchase an additional scratch card with their winnings (*n* = 25). In the loss condition (*n* = 68), 21% (*n* = 14) of participants purchased an additional card, and in the near-miss condition (*n* = 69), 16% (*n* = 11) of participants decided to purchase. A Chi square test of independence revealed that these frequencies were not significantly different from each other, *X*^2^ (1) = 0.50, *p* = .481.

Based on our pre-registered hypotheses and data analysis plan, we also examined relations between participants’ endorsement of the illusion of control and their decision to purchase another card. This point biserial correlation was not significant, *r*(80) = .088, *p* = .435, indicating that there was no association between participant’s illusion of control scores and their purchasing behaviour. In line with this result, a factorial ANOVA examining illusion of control scores with condition and purchase status as between-subjects factors revealed no significant effects (all *p*’s > .1).

### Urge to Continue Gambling

#### Analytical Strategy

**Fig. 1:**
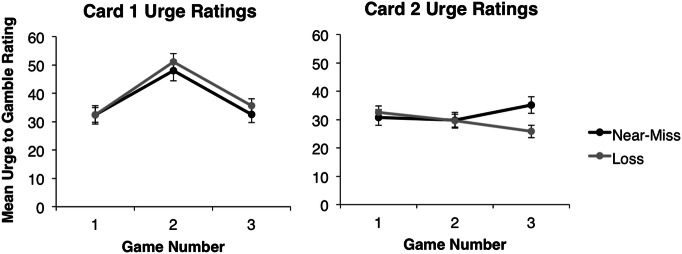
Mean urge to gamble ratings for Card 1 and 2 by condition. Error bars ± 1 SEM

Recall that all participants received an identical sequence of outcomes on card 1 (loss, win of $5.00, loss) but a different sequence of outcomes on card 2 (either loss, loss, loss, or loss, loss, near-miss) with condition assignment based on the type of card 2 participants happened to choose. To examine participants’ urge to continue gambling, average urge ratings for each outcome type were calculated for each condition (those who chose a loss card compared to a near-miss card for card 2). This resulted in six average urge ratings for each condition of our design. Three participants were removed from all urge analyses and three were removed from card 1 urge analyses for giving an incorrect number of ratings per card. Participants’ urge ratings for card 1 and card 2 were compared separately. In the case of violations of sphericity assumptions, degrees of freedom and *F* values are reported with a Greenhouse–Geisser correction. Follow-up comparisons between outcomes were conducted using *t*-tests. Mean urge ratings for both conditions across both cards are depicted in Fig. 1.

#### Card 1

The overall mixed factorial ANOVA for card 1 revealed a significant main effect of outcome, *F*(2, 258) = 105.52, *p* < .001, $\eta_{p}^{2}$ = .45, with no significant main effect of condition or outcome by condition interaction (both *p*’s > .1). Collapsing across condition, paired samples *t*-tests revealed significant differences between urge ratings for game 2 (the winning outcome, *M* = 49.56, *SD* = 26.67) and urge ratings for the losses both before (*M* = 32.40, *SD* = 23.73; *t*[130] = 12.01, *p* < .001) and after (*M* = 34.05, *SD* = 21.75; *t*[130] = 12.42, *p* < .001) this win. No significant differences in urge ratings for the two losses were observed, *t*(130) = 1.34, *p* = .182.

#### Card 2

The overall mixed factorial ANOVA for card 2 revealed no significant main effects of outcome or condition (both *p*’s > .1), but did reveal a significant outcome by condition interaction, *F*(1.56, 205.42) = 11.36, *p* < .001, $\eta_{p}^{2}$ = .08. To identify the source of the interaction, independent *t*-tests between the conditions were conducted at each outcome. These tests revealed no significant differences in urge between conditions at the first outcome, a loss for both conditions (loss condition: *M* = 32.49, *SD* = 18.77; near-miss condition: *M* = 30.73, *SD* = 22.87), *t*(132) = 0.49, *p* = .627, or at the second outcome, also a loss for both conditions (loss condition: *M* = 29.61, *SD* = 18.71; near-miss condition: *M* = 29.84, *SD* = 22.89), *t*(132) = 0.06, *p* = .951. However, there was a significant difference between conditions at the third outcome (loss for the loss condition: *M* = 25.81, *SD* = 17.68; near-miss for the near-miss condition: *M* = 35.19, *SD* = 24.27), *t*(132) = 2.56, *p* = .012.

### Association Between Urge and Purchase Status

**Fig. 2:**
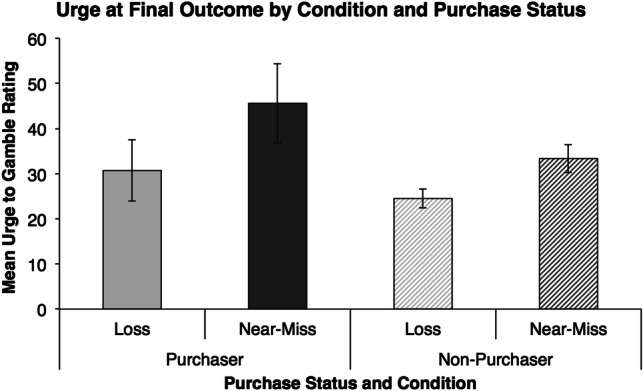
Mean urge to gamble rating following the final scratch card outcome before the decision to purchase. Error bars ± 1 SEM

As outlined in our pre-registered data analysis plan, we conducted a point-biserial correlation to examine the association between urge at the final outcome and purchase status in each of the conditions. In both the loss and near-miss conditions, urge at the final outcome was not associated with purchase status (all *p*’s > .1). An additional test of this association was conducted with a factorial ANOVA examining urge ratings at the final outcome with purchasing status and condition as the between-subjects factors, replicating an analysis in our previous investigation (Stange et al. 2017b) and as outlined in our pre-registration. This analysis revealed a main effect of condition, *F*(1, 30) = 6.10, *p* = .015, $\eta_{p}^{2}$ = .05, and a marginal main effect of purchase status, *F*(1, 30) = 3.68, *p* = .057, $\eta_{p}^{2}$ = .03 (see Fig. 2). However, we did not replicate the purchase status by condition interaction as reported in our previous investigation and as predicted in our pre-registered hypotheses (Stange et al. 2017b).

### Illusion of Control Scores and Purchasing Behaviour

To examine the influence of illusion of control scores on the decision to purchase additional cards, we conducted a *t*-test on illusion of control scores between purchasers and non-purchasers in the near-miss condition. This *t*-test did not reveal a significant difference between purchasers and non-purchasers in terms of illusion of control, *t*(41) = 0.52, *p* = .604. A second pre-registered analysis concerned increases in urge following near-miss outcomes relative to losing outcomes. Within the near-miss condition, we calculated a difference score to examine the change in urge to gamble from the second outcome on card 2 (a loss) to the third outcome on card 2 (the near-miss), and correlated this change in urge with illusion of control scores. This correlation was not significant (*p* = .854), indicating no significant association between the magnitude of urge increases following a near-miss relative to losses and endorsement of the illusion of control.

### Exploratory Analyses

#### Purchasing Behaviour Across Bet Sizes

One goal of the current investigation was to examine the influence of an increased bet size on the decision to purchase additional scratch cards during our experimental paradigm. Given the close procedural similarity between this experiment and our previous investigation, we decided to compare rates of purchasing between the two experiments, the first utilizing a gamble of $2.00 (40% of the participant’s total winnings; Stange et al. 2017b), and the current study utilizing a gamble of $5.00 (100% of the participant’s total winnings). To examine differences in overall levels of purchasing between the samples, a Chi square test of independence was conducted to compare the frequency of purchasers and non-purchasers in the previous study (*n* = 20 purchasers, *n* = 44 non purchasers) to the current study (*n* = 25 purchasers, *n* = 112 non-purchasers). This test revealed a significant difference between experiments, such that rates of purchasing in the current investigation were significantly lower than in the previous study, *X*^2^ (1) = 4.24, *p* =.039.

## Discussion

In an effort to further open science practices within the field of gambling studies, as well as to address issues of replicability in psychological research, we conducted a pre-registered experiment to investigate factors that may influence scratch card purchasing behaviour. In our pre-registration and experimental design, we included a built-in replication of previous findings concerning the influence of specific game outcomes on urge to continue gambling (Stange et al. 2017b), as well as new hypotheses concerning the role of the illusion of control in the impact of near-miss outcomes on behaviour. The current study also utilized a far larger sample size than our previous investigation to ensure adequate power for replication, and this sample size was included in our pre-registration. Although we did not find support for our hypotheses concerning the illusion of control, the results of the current study did replicate our previously reported pattern of urge ratings following specific scratch card outcomes.

The heart of our analyses centered on the influence of specific game outcomes on the urge to continue gambling. Overall, the results of the current study suggest that scratch card outcomes do influence participants’ urge to continue gambling, as our predicted, pre-registered pattern of urge findings was replicated, with wins and near-misses leading to increases in the urge to continue gambling compared to regular losing outcomes. However, we did not replicate our previously reported association between urge ratings at the final outcome and the decision to purchase additional cards in the near-miss condition.

Further, support for nearly all of our pre-registered predictions concerning the role of illusion of control in purchasing decisions was not observed. As near-miss outcomes have been shown to invigorate motivated behaviour across a range of gambling domains (Clark et al. 2009, 2013; Stange et al. 2016), and neural activity to near-miss outcomes has been associated with endorsement of erroneous gambling cognitions as a whole (Clark et al. 2009; Dymond, et al. 2014), we believed that illusion of control may be involved in the processing of near-miss outcomes. If, when uncovering two top prize symbols, a participant sees these symbols as harbingers of a future win, they may be more likely to purchase additional scratch cards. However, we found no difference in illusion of control scores between purchasers and non-purchasers in the near-miss condition as predicted. There was also no association between illusion of control scores and change in urge from a regular loss to the near-miss outcome, a measure of the degree of reactivity created by near-miss outcomes. Therefore, the results of this study do not offer support for the hypothesis that erroneous cognitions related to the illusion of control play a role in scratch card purchasing behaviour, particularly after experiencing a near-miss. It is possible that other gambling related cognitions may interact with the motivational impact of near-miss outcomes to influence gambling decision making, and that including all subscales of the GRCS may have been more informative. Future research should investigate the role of other gambling cognitions in the influence and experience of scratch card near-miss outcomes, such as predictive control and interpretive bias (Billieux et al. 2012).

Although we predicted that more participants in the near-miss condition would purchase additional scratch cards, we observed roughly equal numbers of purchasers in each condition. We observed this despite increasing the cost to purchase an additional card, in an effort to capture purchasing behaviour directly related to changes in subjective experience due to specific outcomes presented in the scratch card games. In our previous study we observed that some participants decided to purchase an additional card despite not reporting an increase in urge; we suggested that this may have been due to some gamblers purchasing on a whim given the relatively low cost of the card that participants could purchase (only risking 40% of their winnings). By increasing the cost to purchase an additional card, we reduced the rates of purchasing overall, but counter to our expectation, this reduction was not specific to those with low urge in the final game of the loss condition. Roughly equal proportions of participants in each condition elected to purchase an additional scratch card, with some purchasing an additional card after experiencing relatively low levels of urge after encountering the loss in the third game of card 2.

When discussing the purchasing behaviour results, it is worth considering the structural aspects of the study that may have played a role. The current purchasing scenario differs substantially from our previous investigation. Here, participants had to risk all of their winnings—$5.00 in total—to get another chance at the top prize. In our previous study, participants only had to risk $2.00 of their $5.00 in winnings (or 40%). In this lower-stakes scenario, one may expect a stronger association between urge at the final outcome and purchasing, as this purchase does not come at a very high cost and may be more strongly influenced by structural factors of the game, such as experiencing a near-miss. Indeed, in our previous investigation we did find a correlation between urge at the final outcome and purchase status, but only for the near-miss condition. However, in the current study we did not find a correlation between urge at this final near-miss outcome and purchasing behaviour suggesting that within the higher-cost purchasing situation, urge at the final outcome was not sufficient to prompt gamblers to risk all of their winnings and purchase an additional card. This higher-cost purchasing scenario leaves open the possibility that a greater amount of urge must be experienced in order for it to factor into purchasing decisions. Perhaps when costs are lower (e.g., participants are foregoing only 40% of their winnings), there is a tighter coupling between moment-to-moment increases in urge due to game features and the decision to purchase. When costs increase, this relation disappears, as suggested by the decreased number of purchasers overall within the current study.

Therefore, although near-misses in scratch cards have been shown to reliably increase the urge to gamble, they are clearly not the defining factor when it comes to the decision to purchase additional scratch cards. What else might factor in to the decision to purchase? The current study suggests that bet size has some influence on this decision, as increasing the cost of another card did significantly reduce purchasing overall. However, there was still no difference in terms of the number of participants who decided to purchase within each outcome condition. While bet size seems to play some role in the decision to purchase, the influence of the effects of individual game outcomes over and above this factor do not seem as impactful, as evidenced by the lack of difference between the near-miss and loss conditions in terms of further scratch card purchasing.

Despite the absence of any influence of condition on purchasing status, it is still abundantly clear that discrete game outcomes *do* impact subjective outcome processing, and in the case of small wins and near-misses, lead to consistent increases in the urge to gamble. Although these increases in urge may not reliably translate into purchasing behaviour within the current paradigm, it is worth noting that these effects are being measured after experiencing only one near-miss. Real scratch cards tend to contain multiple near-misses on each individual card (Stange et al. 2016), and foundational research examining the impact of slot machine near-miss outcomes on gambling behaviour studied the impact of many such near-miss outcomes on the decision to continue gambling (Kassinove and Schare 2001). Therefore, perhaps experiencing *multiple* near-miss events within a session is necessary to exert an appreciable influence on the decision to continue to gamble. For example, previous slot machine studies have reported that gambling behaviour was most impacted by near-miss outcomes when the percentage of spins containing them within a gambling session was 30% (Kassinove and Schare 2001). Future research should examine the effect of multiple scratch card near-miss outcomes on gambler experience and behaviour to clarify these associations.

Finally, when considering the results of the current experiment, it is interesting that self-reported increases in the urge to continue gambling following near-miss outcomes are not *more* closely related to the decision to purchase additional cards. In the current study, we did see a marginal effect of purchasing status on urge at the final game outcome, but we did not replicate our previous finding which showed that urge significantly predicted the decision to purchase after experiencing a near-miss in the final game, but not after experiencing a loss in the final game. Although we speculate that our inability to redemonstrate this relationship may be related to the increase in card cost in the current experiment, this finding also highlights the notion that if the decision to purchase is not entirely based on situational, outcome-level influences, perhaps there is an individual difference that makes some participants more likely to purchase additional cards *regardless* of their experiences during the game. That is, regardless of their responses to specific outcomes. The current results suggest that perhaps individuals who purchase additional cards are more reactive to scratch card outcomes in general (regardless of the outcome), as evidenced through the (marginal) main effect of purchase status on urge at the final outcome. Additionally, even though rates of purchasing decreased in the current investigation from our previous study, there were still participants who elected to purchase an additional card. Future research should attempt to determine what other situational factors or individual difference variables may be influential in these decision-making processes.

## Limitations

The current study has a few key limitations. First, we utilized a sample of undergraduate students, the majority of whom had only purchased scratch cards one to five times in the past 12 months (see Table 1). Although our sample did consist entirely of participants who had experience with scratch card games, it’s possible that sampling from a population with greater variation in their levels of experience may have yielded different results. Relatedly, a sample recruited on the basis of scratch card gambling frequency would allow us to determine if the frequency of scratch card gambling plays a role in experienced urge to gamble and subsequent purchasing behaviour. For example, motives to continue gambling may differ between frequent and infrequent gamblers. A related concern is the range of illusion of control scores within our sample. Given the lower levels of gambling frequency, and lower levels of self-reported problem gambling symptomatology, it is perhaps unsurprising that our sample also consisted of individuals who did not greatly endorse the illusion of control erroneous cognitions subscale. This relatively restricted range of responses may have impeded our ability to assess differences in purchasing behaviour based on these scores. To address these limitations, future studies could utilize community recruitment methods, allowing for a more representative sampling of scratch card gamblers.

An additional limitation that is commonly encountered with laboratory-based gambling scenarios concerns the fact that participants were not gambling with their own money during the experiment, as they received their first two scratch cards for free. However, if participants decided to purchase an additional card, they did have to forego their own winnings and use this money for the opportunity to gamble. Nevertheless, it’s possible that this monetary gain that all participants encountered on their first card is experienced differently when an initial cost has been incurred (i.e., one purchases a card initially), compared to when it is encountered without an upfront cost (i.e., a scratch card is given to you for free). How individuals approach these types of financial decisions may have had an influence on participants’ willingness to spend their winnings on an additional card.

## Conclusion

The current study adds to a small but growing body of gambling literature examining the subjective and behavioural consequences of various outcomes encountered during scratch card gambling. Although we did not observe any effects of outcome or the endorsement of the illusion of control on purchasing behaviour, we did replicate previous results in terms of the influence of near-miss outcomes on participants’ urge to continue gambling. In the current study we found that overall rates of purchasing were lower than in previous investigations, likely due to the higher cost, and hence riskier nature of the gamble presented. Within the current purchasing paradigm, we did not find an association between urge at the final outcome and the decision to purchase additional cards in the near-miss condition, as observed in the past. It is possible that the higher-stakes gamble we presented to participants in the current study precluded participants from acting on the increase in urge created by near-misses. Instead, the decision to purchase may have been driven by an unknown trait-level construct. In conclusion, future research should investigate other candidate individual differences that may impact the decision to purchase additional scratch card games, as well as different manipulations of the purchasing decision itself, in order to better understand how individual differences, structural characteristics, and decision making processes interact to influence scratch card gambling behaviour.

## Footnotes

1 A sampling of Ontario Lottery and Gaming Corporation scratch cards at the time of writing confirms this, with prizes equivalent to the value of the card (e.g., a push) the most common prize in a sample card from each of the card price categories: $1 (OLG 2019a), $3 (OLG 2019b), $4 (OLG 2019c), $5 (OLG 2019d), and $20 (OLG 2019e).
